# NOTCH3 missense mutations as predictor of long-term response to gemcitabine in a patient with epithelioid hemangioendothelioma

**DOI:** 10.1007/s00432-023-04598-1

**Published:** 2023-02-07

**Authors:** Moritz Schmidt, Sven Mattern, Stephan Singer, Martin Schulze, Saskia Biskup, Patrick Krumm, Ulrich M. Lauer, Lars Zender, Clemens Hinterleitner, Martina Hinterleitner

**Affiliations:** 1 Department of Medical Oncology and Pneumology (Internal Medicine VIII), University Hospital Tuebingen, 72076 Tuebingen, Germany; 2 DFG Cluster of Excellence 2180 ‘Image-Guided and Functional Instructed Tumor Therapy’ (iFIT), University of Tuebingen, 72076 Tuebingen, Germany; 3 Department of Pathology, University Hospital Tuebingen, Tuebingen, Germany; 4 Praxis Für Humangenetik Tübingen, Tuebingen, Germany; 5 CeGaT GmbH, Center for Genomics and Transcriptomics, Tuebingen, Germany; 6 Department of Radiology, University Hospital Tuebingen, Tuebingen, Germany; 7 German Cancer Consortium (DKTK), German Cancer Research Center (DKFZ), Tuebingen, Germany; 8 Cancer Biology and Genetics, Memorial Sloan Kettering Cancer Center, New York, NY USA

**Keywords:** Epithelioid hemangioendothelioma, NOTCH3, Gemcitabine, Soft-tissue sarcoma

## Abstract

**Purpose:**

Epithelioid hemangioendothelioma (EHE) as a very rare malignant vascular tumor belongs to the heterogenous group of soft-tissue sarcomas. Depending on the clinical course of the disease, interdisciplinary treatment concepts are required, including surgery, radiotherapy and systemic cancer therapy. However, due to its uncommonness, standard treatment options are lacking so far, especially in advanced disease with distant metastases.

**Methods and results:**

Here we report on an unusual case of a patient with metastasized EHE showing long-term response to second line treatment with gemcitabine over almost 2 decades. Cancer genome sequencing of the patient’s tumor tissue detected a NOTCH3 missense mutation which could provide an explanation for these clinical findings. NOTCH3 is known to be a mediator of resistance towards gemcitabine-based cancer treatment, at least in pancreatic cancer and non-small cell lung cancer.

**Conclusion:**

The observation that this missense mutation of NOTCH3 is associated with an increased response to treatment with gemcitabine in EHE can be used prospectively to assess NOTCH3 as potential biomarker for predicting therapy response to gemcitabine.

Epithelioid hemangioendothelioma (EHE) represents a rare entity of soft-tissue sarcoma (STS) with a prevalence of < 1/1,000,000, mainly affecting young and middle-aged adults (de Pinieux et al. 2021; Flucke et al. 2014; Guo et al. 2017; Lau et al. 2011; Rosenbaum et al. 2020; Stacchiotti et al. 2021a, b). As a malignant vascular tumor, the disease originates from endothelial or pre-endothelial cells and can manifest in any part of the body, with the lung being the most frequent location (30% of all cases) (Lau et al. 2011; Sardaro et al. 2014). The clinical course varies from low malignant to highly aggressive disease with the tendency of rapid distant metastasis (Stacchiotti et al. 2021a, b). The diagnosis is often made incidentally during routine imaging. However, depending on the site of manifestation, patients may also be alerted to the disease by general symptoms such as weight loss and anemia, respiratory symptoms, bone pain or neurologic abnormalities which are associated with a worse prognosis (Amin et al. 2006; Bagan et al. 2006; Kitaichi et al. 1998). Histologically, EHE is defined via accumulation of epithelioid endothelial tumor cells embedded in a myxohyaline or fibrous stroma (Fig. 1a). Of note, diagnosis of EHE can be challenging as it shares common histopathological features of several carcinomas, sarcomas (especially epithelioid and angiosarcoma) as well as mesothelioma or even autoimmune diseases like sarcoidosis (Anderson et al. 2015; Rosenberg and Agulnik 2018; Stacchiotti et al. 2021a, b).

**Fig. 1:**
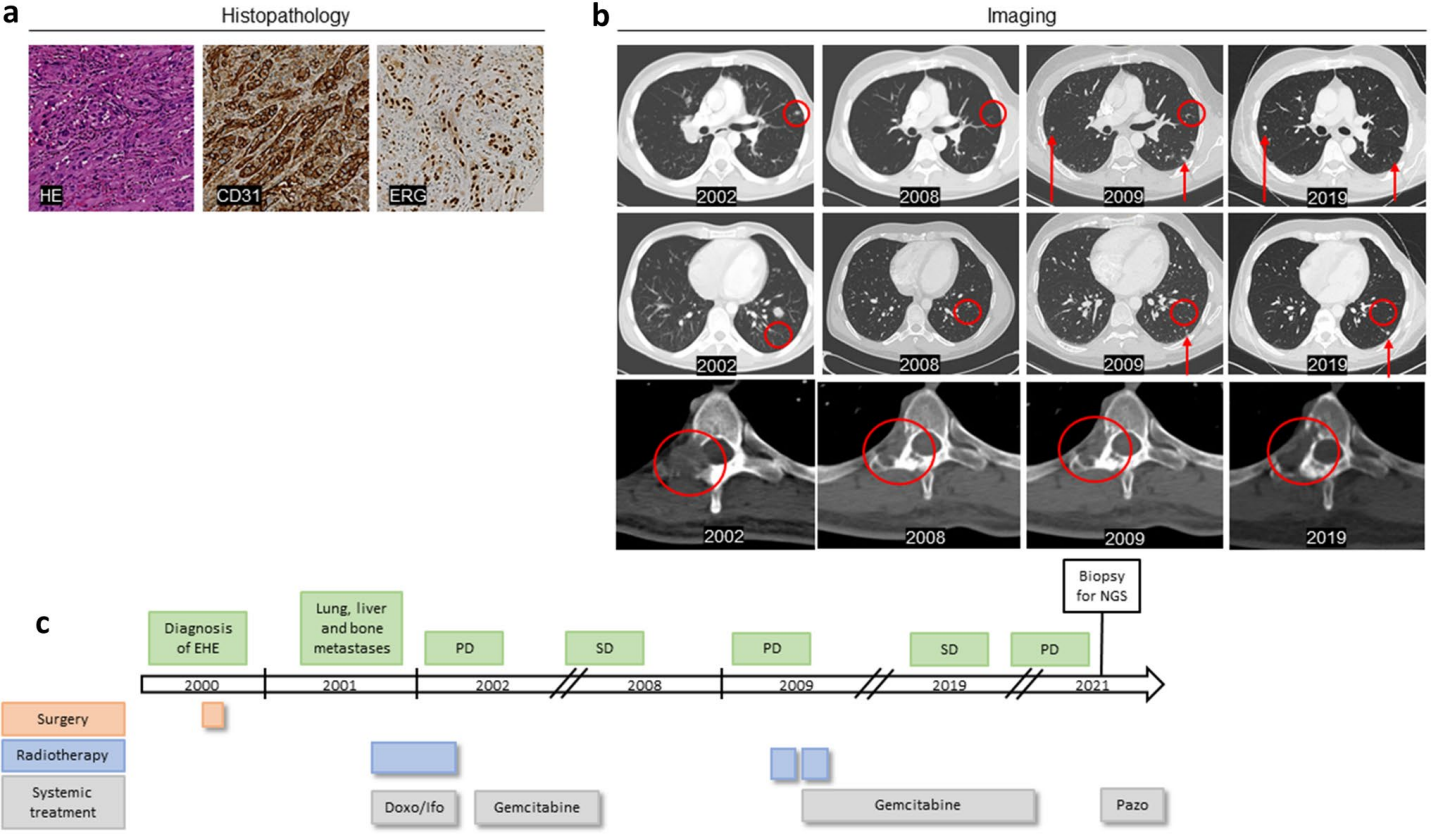
Clinical and histopathological presentation of the EHE patient and treatment scheme. **a** Histopathology of a bone metastasis localized within the left os ilium, performed at April 2017, shows epithelioid tumor cells embedded in a fibrous stroma (HE) with strong expression of endothelial markers CD31 (membranous) and ERG (nuclear). **b** Assessment of exemplary pulmonary and bone lesions via CT scan in the period between 2002 and 2019. Newly detected lesions are indicated by red arrows. **c** Multimodal treatment scheme of this EHE patient between 2000 and 2021. (PD, progressive disease; *SD* stable disease, *Doxo* doxorubicin, *Ifo* ifosfamide, *NGS* next-generation sequencing, *Pazo* pazopanib)

A common genetic alteration of EHE is the occurrence of specific gene fusions, including WWTR1-CAMTA1, being the most frequently detected translocation in EHE, or YAP1-TFE3 (Antonescu et al. 2013; Errani et al. 2011). These genetic aberrations have been discussed to contribute to pathophysiology of EHE since both WWTR1 and YAP1 represent central effector proteins of the Hippo pathway mediating pro-oncogenic transcriptional effects (Merritt et al. 2021; Salem and Hansen 2019; Wu and Yang 2018; Zender et al. 2006).

For patients with limited disease, tumor resection shows curation rates of 70 up to 80% (Rosenbaum et al. 2020; Stacchiotti et al. 2021a, b). In metastatic disease, however, no standard therapy is established so far and treatment options are still very limited. Retrospective analyses investigating several different chemotherapeutic agents, small molecule inhibitors and immunomodulatory agents showed only limited response rates (Sardaro et al. 2014). Exemplarily, a retrospective international case series showed a progression-free survival of no more than 5 months and no partial response in a cohort of patients with advanced EHE under systemic treatment with doxorubicin and ifosfamide (Frezza et al. 2021).

Here, we present an unusual and rare case of a metastasized EHE with a long-term response over 205 months (17 years) to a second line systemic treatment with gemcitabine after initial tumor resection. Our case refers to a casuistic of EHE partially displayed in a previous report by Pintoffl et al. (Pintoffl et al. 2009). At this time point, the authors described the unique course of this case under systemic treatment with gemcitabine over a period of 72 months. Referring to this, almost 14 years later, we provide an update on this very unusual case and relate the therapeutic response to meanwhile available molecular features of the tumor tissue.

In June 2000, a 30-year-old man was diagnosed with EHE of the right scapula. After computed tomography (CT) scan-based exclusion of distant metastases a complete tumor excision was performed by means of a Tikhoff–Linberg procedure type IV (Voggenreiter et al. 1999). In November 2001, a subsequent CT scan showed multiple lung, liver and bone metastases (Fig. 1b). Between November 2001 and February 2002, the patient underwent both local radiotherapy of multiple osteolytic lesions and systemic treatment with doxorubicin (25 mg/m^2^) in combination with ifosfamide (2 g/m^2^), each compound applied for three consecutive days, given for four therapy cycles with a cycle length of 21 days, respectively, in total.

After a subsequent disease progression in March 2002, a systemic second-line treatment with gemcitabine (administered with 1 g/m^2^ on days 1, 8 and 15 of each therapy cycle comprising a total of 28 days) was initiated. Between March 2002 and January 2008, the patient received in total 62 therapy cycles leading to a stabilization of the disease with even partial regression of the pulmonary lesions. Subsequently, treatment with gemcitabine was discontinued. In June 2009, a general tumor progression was observed. As a result, a stereotactic irradiation of two unstable lumbar osteolytic lesions as well as of newly detected cerebral metastases was combined with a systemic treatment, again employing gemcitabine. Gemcitabine treatment was consequently administered for a total of 164 therapy cycles until January 2021. Remarkably, in that time period no further tumor progression was observed. The patient showed an excellent overall tolerability without any signs of long-term toxicities enabling an unrestricted participation in everyday life (ECOG performance status grade 0). The patient was fully employed and physically active under systemic treatment with gemcitabine and had no need of supportive medication. In February 2021, detection of further systemic tumor progression led to treatment change to pazopanib (Fig. 1c).

The present case highlights the potential benefit of a systemic treatment with gemcitabine in EHE. Gemcitabine as an anti-metabolite mediates its cytotoxic effects via integration into the DNA of tumor cells leading to an abortion of DNA synthesis (Plunkett et al. 1995). To our knowledge, only case series of patients with advanced EHE treated with gemcitabine, mostly in combination with docetaxel, are reported (Frezza et al. 2021; Sabile et al. 2021; Zhou et al. 2020). According to these publications, systemic treatment of EHE with gemcitabine, at least in combination with docetaxel, could lead to a stabilization of the disease in some patients. However, long-term response to therapy with single treatment of gemcitabine is rarely described so far. Furthermore, in most cases, an assessment of the genetic profile of the tumor has not been performed.

Our patient presented a YAP1-TFE3 fusion which has been described to act as tumor-driving gene fusion in a subset of EHE (Antonescu et al. 2013; Rosenbaum et al. 2020; Szulzewsky et al. 2021). Previous studies revealed the significance of YAP1 and TFE3 for carcinogenesis in several tumor entities, such as liver cancer, breast cancer, prostate cancer or renal cancer (Salem and Hansen 2019; Wu and Yang 2018; Yin et al. 2019; Zender et al. 2006). Of note, overactivation of Hippo/YAP1 signaling is known to be associated with resistance to anti-metabolites, like gemcitabine, at least in pancreatic and colorectal cancer (Nguyen and Yi 2019). However, the impact of YAP1-TFE3 fusion on response to gemcitabine in EHE is not investigated so far.

Interestingly, further somatic molecular genetic analyses of a pelvic bone metastasis (Ki-67 proliferation index of 5%) in 2021 by means of the Illumina HiSeq/NovaSeq system and a 750-gene next-generation sequencing panel identified somatic mutations of PTK7, RPTOR as well as a heterozygous NOTCH3 missense mutation (p.Asn1964Ser). To our knowledge, the significance of this NOTCH3 missense mutation has not been described in the literature. However, protein domain analyses revealed that the abovementioned missense mutation affects ankyrin repeat domains of NOTCH3 that are crucially involved in mediating protein–protein interactions and alterations in these domains cause functional impairment and lead to disease (Li et al. 2006). It could be shown that NOTCH3 is involved in mediating resistance to gemcitabine in pancreatic and non-small cell lung cancer (Hu et al. 2018; Xiu et al. 2021). In vitro studies revealed that NOTCH3 promotes resistance to gemcitabine via activation of PI3K/Akt signaling in pancreatic cancer (Yao and Qian 2010). Additionally, NOTCH3 could be identified as parameter for predicting therapy response to gemcitabine in pancreatic cancer: expression of NOTCH3 inversely correlated with therapy response and overall survival of patients treated with gemcitabine (Eto et al. 2013). As a result, siRNAs or γ-secretase inhibitors targeting NOTCH3 are currently under investigation to overcome therapy resistance and sensitize pancreatic and non-small cell lung cancer to treatment with gemcitabine (Hu et al. 2018; Yao and Qian 2010). The role of NOTCH3 in mediating chemoresistance in EHE remains unclear so far.

The clinical findings presented in this case provide first evidence for a relevance of NOTCH3 in the context of gemcitabine-based treatment of EHE. Our case highlights the potential benefit of a monotherapy with gemcitabine in advanced EHE with a tolerable toxicity profile. Since genetic analyses from EHE patients previously treated with gemcitabine are lacking so far, we here show a potential correlation between the therapy response to gemcitabine and the presence of a NOTCH3 missense mutation. Further prospective clinical trials are needed to investigate the role of gemcitabine as systemic treatment option in a larger cohort of patients with advanced EHE and to further assess NOTCH3 as potential biomarker for predicting therapy response to gemcitabine.

## Data Availability

The data supporting our findings are available from the corresponding author upon reasonable request.

## References

[CR1] Amin RM, Hiroshima K, Kokubo T, Nishikawa M, Narita M, Kuroki M, Nakatani Y (2006) Risk factors and independent predictors of survival in patients with pulmonary epithelioid haemangioendothelioma. Review of the literature and a case report. Respirology 11(6):818–825. 10.1111/j.1440-1843.2006.00923.x 17052315 10.1111/j.1440-1843.2006.00923.x

[CR2] Anderson T, Zhang L, Hameed M, Rusch V, Travis WD, Antonescu CR (2015) Thoracic epithelioid malignant vascular tumors: a clinicopathologic study of 52 cases with emphasis on pathologic grading and molecular studies of WWTR1-CAMTA1 fusions. Am J Surg Pathol 39(1):132–139. 10.1097/PAS.0000000000000346 25353289 10.1097/PAS.0000000000000346 PMC4268225

[CR3] Antonescu CR, Le Loarer F, Mosquera JM, Sboner A, Zhang L, Chen CL, Fletcher CD (2013) Novel YAP1-TFE3 fusion defines a distinct subset of epithelioid hemangioendothelioma. Genes Chromosomes Cancer 52(8):775–784. 10.1002/gcc.22073 23737213 10.1002/gcc.22073 PMC4089994

[CR4] Bagan P, Hassan M, Le Pimpec Barthes F, Peyrard S, Souilamas R, Danel C, Riquet M (2006) Prognostic factors and surgical indications of pulmonary epithelioid hemangioendothelioma: a review of the literature. Ann Thorac Surg 82(6):2010–2013. 10.1016/j.athoracsur.2006.06.068 17126100 10.1016/j.athoracsur.2006.06.068

[CR5] de Pinieux G, Karanian M, Le Loarer F, Le Guellec S, Chabaud S, Terrier P, French Sarcoma Group- Groupe d’Etude des Tumeurs Osseuses (2021) Nationwide incidence of sarcomas and connective tissue tumors of intermediate malignancy over four years using an expert pathology review network. PLoS ONE 16(2):e0246958. 10.1371/journal.pone.0246958 33630918 10.1371/journal.pone.0246958 PMC7906477

[CR6] Errani C, Zhang L, Sung YS, Hajdu M, Singer S, Maki RG, Antonescu CR (2011) A novel WWTR1-CAMTA1 gene fusion is a consistent abnormality in epithelioid hemangioendothelioma of different anatomic sites. Genes Chromosomes Cancer 50(8):644–653. 10.1002/gcc.20886 21584898 10.1002/gcc.20886 PMC3264678

[CR7] Eto K, Kawakami H, Kuwatani M, Kudo T, Abe Y, Kawahata S, Sakamoto N (2013) Human equilibrative nucleoside transporter 1 and Notch3 can predict gemcitabine effects in patients with unresectable pancreatic cancer. Br J Cancer 108(7):1488–1494. 10.1038/bjc.2013.108 23492684 10.1038/bjc.2013.108 PMC3629422

[CR8] Flucke U, Vogels RJ, de Saint Aubain N, Somerhausen CDH, Riedl RG, van Gorp JM, Mentzel T (2014) Epithelioid Hemangioendothelioma: clinicopathologic, immunohistochemical, and molecular genetic analysis of 39 cases. Diagn Pathol 9:131. 10.1186/1746-1596-9-131 24986479 10.1186/1746-1596-9-131 PMC4100035

[CR9] Frezza AM, Ravi V, Lo Vullo S, Vincenzi B, Tolomeo F, Chen TW, Stacchiotti S (2021) Systemic therapies in advanced epithelioid haemangioendothelioma: a retrospective international case series from the world sarcoma network and a review of literature. Cancer Med 10(8):2645–2659. 10.1002/cam4.3807 33713582 10.1002/cam4.3807 PMC8026938

[CR10] Guo Q, Xue J, Xu L, Shi Z, Zhou B (2017) The clinical features of epithelioid hemangioendothelioma in a Han Chinese population: a retrospective analysis. Medicine (baltimore) 96(26):e7345. 10.1097/MD.0000000000007345 28658152 10.1097/MD.0000000000007345 PMC5500074

[CR11] Hu BD, Guo J, Ye YZ, Du T, Cheng CS, Jiang Q, Zhang YB (2018) Specific inhibitor of Notch3 enhances the sensitivity of NSCLC cells to gemcitabine. Oncol Rep 40(1):155–164. 10.3892/or.2018.6448 29781034 10.3892/or.2018.6448 PMC6059738

[CR12] Kitaichi M, Nagai S, Nishimura K, Itoh H, Asamoto H, Izumi T, Dail DH (1998) Pulmonary epithelioid haemangioendothelioma in 21 patients, including three with partial spontaneous regression. Eur Respir J 12(1):89–96. 10.1183/09031936.98.12010089 9701420 10.1183/09031936.98.12010089

[CR13] Lau K, Massad M, Pollak C, Rubin C, Yeh J, Wang J, Weinberg G (2011) Clinical patterns and outcome in epithelioid hemangioendothelioma with or without pulmonary involvement: insights from an internet registry in the study of a rare cancer. Chest 140(5):1312–1318. 10.1378/chest.11-0039 21546438 10.1378/chest.11-0039

[CR14] Li J, Mahajan A, Tsai MD (2006) Ankyrin repeat: a unique motif mediating protein-protein interactions. Biochemistry 45(51):15168–15178. 10.1021/bi062188q 17176038 10.1021/bi062188q

[CR15] Merritt N, Garcia K, Rajendran D, Lin ZY, Zhang X, Mitchell KA, Tanas MR (2021) TAZ-CAMTA1 and YAP-TFE3 alter the TAZ/YAP transcriptome by recruiting the ATAC histone acetyltransferase complex. Elife. 10.7554/eLife.62857 33913810 10.7554/eLife.62857 PMC8143797

[CR16] Nguyen CDK, Yi C (2019) YAP/TAZ signaling and resistance to cancer therapy. Trends Cancer 5(5):283–296. 10.1016/j.trecan.2019.02.010 31174841 10.1016/j.trecan.2019.02.010 PMC6557283

[CR17] Pintoffl J, Meisinger I, Mayer F, Horger M, von Weyhern C, Kanz L, Hartmann JT (2009) Long-term disease stabilization during second-line gemcitabine in a refractory metastatic haemangioendothelioma. Anticancer Drugs 20(1):73–74. 10.1097/CAD.0b013e32831b9b0b 19343003 10.1097/CAD.0b013e32831b9b0b

[CR18] Plunkett W, Huang P, Xu YZ, Heinemann V, Grunewald R, Gandhi V (1995) Gemcitabine: metabolism, mechanisms of action, and self-potentiation. Semin Oncol 22(4 Suppl 11):3–10 7481842

[CR19] Rosenbaum E, Jadeja B, Xu B, Zhang L, Agaram NP, Travis W, Antonescu CR (2020) Prognostic stratification of clinical and molecular epithelioid hemangioendothelioma subsets. Mod Pathol 33(4):591–602. 10.1038/s41379-019-0368-8 31537895 10.1038/s41379-019-0368-8 PMC7228463

[CR20] Rosenberg A, Agulnik M (2018) Epithelioid hemangioendothelioma: update on diagnosis and treatment. Curr Treat Options Oncol 19(4):19. 10.1007/s11864-018-0536-y 29546487 10.1007/s11864-018-0536-y

[CR21] Sabile JMG, Stump MS, Fitzpatrick FC, Skelton MR (2021) Primary bone marrow epithelioid hemangioendothelioma treated with gemcitabine and docetaxel. JCO Oncol Pract 17(2):118–120. 10.1200/OP.20.00360 32991259 10.1200/OP.20.00360

[CR22] Salem O, Hansen CG (2019) The hippo pathway in prostate cancer. Cells 8(4):370. 10.3390/cells8040370 31018586 10.3390/cells8040370 PMC6523349

[CR23] Sardaro A, Bardoscia L, Petruzzelli MF, Portaluri M (2014) Epithelioid hemangioendothelioma: an overview and update on a rare vascular tumor. Oncol Rev 8(2):259. 10.4081/oncol.2014.259 25992243 10.4081/oncol.2014.259 PMC4419652

[CR24] Stacchiotti S, Frezza AM, Blay JY, Baldini EH, Bonvalot S, Bovee J, Trama A (2021a) Ultra-rare sarcomas: a consensus paper from the connective tissue oncology society community of experts on the incidence threshold and the list of entities. Cancer 127(16):2934–2942. 10.1002/cncr.33618 33910263 10.1002/cncr.33618 PMC8319065

[CR25] Stacchiotti S, Miah AB, Frezza AM, Messiou C, Morosi C, Caraceni A, Gronchi A (2021b) Epithelioid hemangioendothelioma, an ultra-rare cancer: a consensus paper from the community of experts. ESMO Open 6(3):100170. 10.1016/j.esmoop.2021.100170 34090171 10.1016/j.esmoop.2021.100170 PMC8182432

[CR26] Szulzewsky F, Holland EC, Vasioukhin V (2021) YAP1 and its fusion proteins in cancer initiation, progression and therapeutic resistance. Dev Biol 475:205–221. 10.1016/j.ydbio.2020.12.018 33428889 10.1016/j.ydbio.2020.12.018 PMC8107117

[CR27] Voggenreiter G, Assenmacher S, Schmit-Neuerburg KP (1999) Tikhoff-Linberg procedure for bone and soft tissue tumors of the shoulder girdle. Arch Surg 134(3):252–257. 10.1001/archsurg.134.3.252 10088563 10.1001/archsurg.134.3.252

[CR28] Wu L, Yang X (2018) Targeting the hippo pathway for breast cancer therapy. Cancers (basel) 10(11):422. 10.3390/cancers10110422 30400599 10.3390/cancers10110422 PMC6266939

[CR29] Xiu M, Wang Y, Li B, Wang X, Xiao F, Chen S, Hua F (2021) The role of Notch3 signaling in cancer stemness and chemoresistance: molecular mechanisms and targeting strategies. Front Mol Biosci 8:694141. 10.3389/fmolb.2021.694141 34195229 10.3389/fmolb.2021.694141 PMC8237348

[CR30] Yao J, Qian C (2010) Inhibition of Notch3 enhances sensitivity to gemcitabine in pancreatic cancer through an inactivation of PI3K/Akt-dependent pathway. Med Oncol 27(3):1017–1022. 10.1007/s12032-009-9326-5 19816816 10.1007/s12032-009-9326-5

[CR31] Yin X, Wang B, Gan W, Zhuang W, Xiang Z, Han X, Li D (2019) TFE3 fusions escape from controlling of mTOR signaling pathway and accumulate in the nucleus promoting genes expression in Xp11.2 translocation renal cell carcinomas. J Exp Clin Cancer Res 38(1):119. 10.1186/s13046-019-1101-7 30849994 10.1186/s13046-019-1101-7 PMC6408813

[CR32] Zender L, Spector MS, Xue W, Flemming P, Cordon-Cardo C, Silke J, Lowe SW (2006) Identification and validation of oncogenes in liver cancer using an integrative oncogenomic approach. Cell 125(7):1253–1267. 10.1016/j.cell.2006.05.030 16814713 10.1016/j.cell.2006.05.030 PMC3026384

[CR33] Zhou X, Li P, Gu X, Zheng F, Zhao J, Zhao L (2020) A case report of right atrial epithelioid hemangioendothelioma with multiple pulmonary metastases. Clin Respir J 14(2):173–178. 10.1111/crj.13121 31794151 10.1111/crj.13121

